# Association of length of service and job category with occupational health literacy of port employees in Shenzhen, China

**DOI:** 10.1186/s12889-023-15769-7

**Published:** 2023-06-23

**Authors:** Jinlin Wang, Chunbao Mo, Qiujie Sheng, Yuehong Huang, Dafeng Lin, Yuan Liang, Naixing Zhang

**Affiliations:** 1 Shenzhen Prevention and Treatment Center for Occupational Diseases, Shenzhen, 518020 Guangdong China; 2 School of medicine, Southern University of Science and Technology, Shenzhen, 518055 Guangdong China; 3 Shenzhen Chiwan Port Development Co., Ltd, Shenzhen, 518000 Guangdong China; 4 Department of Social Medicine and Health Management, School of Public Health, Tongji Medical College, Huazhong University of Science and Technology, Wuhan, 430030 China

**Keywords:** Length of service, Job category, Occupational health literacy, Port employees

## Abstract

**Background:**

Health literacy (HL) is associated with health outcomes, but little is known about the occupational HL (OHL) for port employees and its link to the length of service and job category.

**Method:**

A cross-sectional survey was conducted on 3492 port employees from the Occupational Health Survey for Port Employees project, and a special questionnaire was utilized to measure the OHL status. Binary and ordinal logistic regressions were used to estimate the association.

**Result:**

Among the participants, 72.90% had sufficient OHL with a mean score (standard deviation) of 53.10 (7.26). Binary logistic regression results indicated that the association between length of service (33–40 years group *Adjusted OR* = 1.11; 41–49 years group *Adjusted OR* = 1.14; ≥50 years group *Adjusted OR* = 1.19) and job category (longshoremen *Adjusted OR* = 0.90; driver *Adjusted OR* = 0.91) with OHL were statistically significant. Ordinal logistic regression results indicated that, for OHL, *Adjusted OR* was increased in different lengths of service level (33–40 years group, *Adjusted OR* = 1.50; 41–49 years group, *Adjusted OR* = 1.75; ≥50 years group, *Adjusted OR* = 2.19), and the *Adjusted OR* of skilled workers was 1.60.

**Conclusion:**

Most port participants had sufficient OHL, and the length of service and job category could affect OHL. The effect of the length of service may be more obvious; the length of service can promote the improvement of OHL continuously.

**Supplementary Information:**

The online version contains supplementary material available at 10.1186/s12889-023-15769-7.

## Introduction

In economic globalization, seaports play an increasingly critical role as the center of ocean transportation along with the development of international trade [1]. According to statistics, among the European Union’s 22 maritime member states, over 110,000 port workers are working in the loading and unloading of ships [2]. Shenzhen Port, the third largest in the world in terms of throughput, employs tens of thousands of people. Given that the nature of work in port is complex, employees are usually exposed to various occupational hazards that seriously threaten their health [3]. Improving the health of port employees is in people’s best interest and for the needs of global health.

Health literacy (HL) is linked to literacy and entails people’s knowledge, motivation, and competence to access, understand, appraise, and apply health information to make decisions about healthcare, disease prevention, and health promotion in their everyday lives to maintain or improve their quality of life [4]. Generally, improving HL is the first step in protecting the occupational population from disease and accidental occupational injury [5]. Low HL is associated with increased morbidity, mortality, and hospitalization, whereas high HL can produce good social benefits [6–8]. Therefore, improving HL has become important in obtaining good health benefits. In the ninth Global Conference on Health Promotion held by the WHO in Shanghai, China, HL, health governance, and health places were considered the three major areas of health promotion [9]. Moreover, the improvement of residents’ HL levels is also regarded as one of the goals of Healthy China 2030 [10]. Since 2012, with the implementation of standardized HL monitoring [11], many studies have focused on evaluating residents’ HL, exploring the influencing factors of HL, and estimating the association between HL and diseases or health outcomes [12]. However, research on occupational HL (OHL) is still rare, especially for port employees. In addition to facing the same health problems, the occupational population faces health threats from various complex occupational hazards in the working environment. Therefore, the OHL situation reflected by the HL scale of the general population is not comprehensive. Moreover, a clear definition of OHL is still absent, and the evaluation method needs to be unified [13]. Thus, a special evaluation program for OHL must be formulated.

In the present study, a special survey scale for port employees was developed, referencing The National Health Literacy Monitoring Questionnaire (2014 edition, NHLMQ) [14, 15], and utilized for the investigation to understand the OHL status of employees. In addition, the scale was used to assess the association between length of service or job category with OHL and provide a basis for making occupational health promotion programs. Numerous previous studies have confirmed the relationship between HL (or OHL) and some demographic characteristics, such as age, sex, and education level [14, 16, 17]. This study assesses the relationship between OHL and other factors, such as length of service and job category. We defined “length of service” as the amount of time an employee has worked in a port and “job category” as different types or categories of jobs based on job content, skill requirements, and work environment in a port, such as longshoreman, repairman, wireman, and welder. Therefore, length of service and job category were the main independent variables in this study, which could clarify the relationship between them and OHL and reflect the effect of occupational health promotion from other perspectives.

## Method

### Study design and participants

**Fig. 1:**
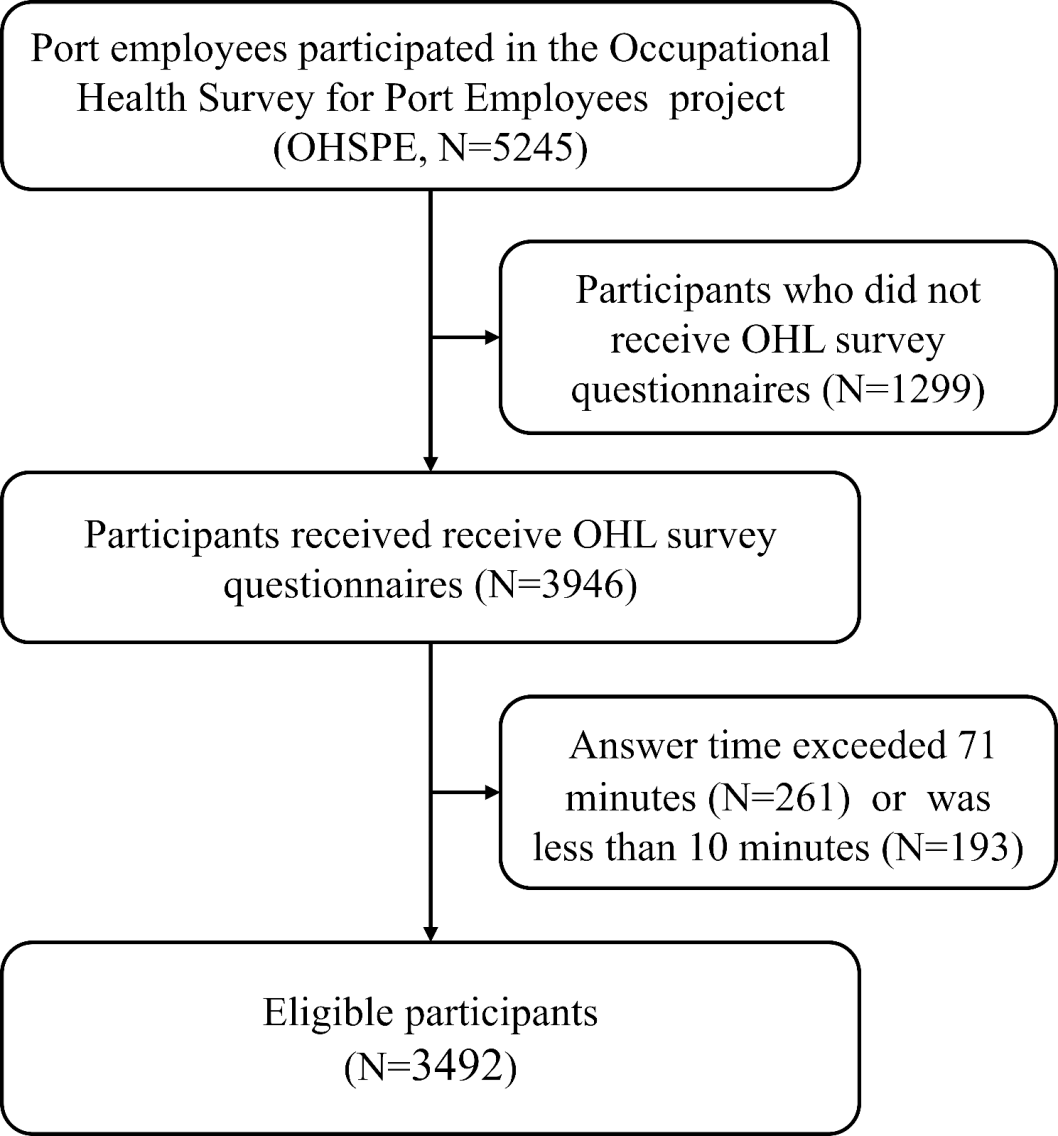
Flow chart of sample selection. OHL, occupational health literacy

The participants were recruited from the Occupational Health Survey for Port Employees project [18]. A total of 5245 port employees (≥ 18 years of age) in Shenzhen, China were enrolled by cluster sampling between June and October 2020. The Shenzhen Prevention and Treatment Center for Occupational Diseases (SPTCOD) implemented the occupational health examination and questionnaires. This project aimed to know the health conditions, evaluate occupational hazards exposure, and assess the effectiveness of occupational health promotion [18]. All the participants must meet the following inclusion criteria: (1) employed in a port of Shenzhen, (2) has worked for more than one year, and (3) understood the purpose and significance of the study and voluntarily signed the informed consent. The exclusion criteria were (1) non-port workers and (2) unwilling to cooperate with investigators. A mandatory mechanism was established to prevent missing values in the questionnaire. In the present study, we excluded 1299 individuals who did not receive OHL survey questionnaires. Additionally, we excluded individuals who took more than 95% (71 min) or less than 5% (10 min) of the answer time of the total population (*Time*_*total*_) as these individuals spent too much time (n = 261) or too little time (n = 193) on this survey. Ultimately, we included 3492 participants for further analysis (Fig. 1). Our sample size was fully adequate for the study, as shown by our estimation (Supplementary material: Sample size estimation). This study complied with the Helsinki Declaration and was approved by the Medical Ethics Committee of SPTCOD (No. LL2020-34).

### Questionnaires

Uniformly trained investigators distributed structured electronic questionnaires to the participants, which they filled in to collect their basic information and assess their OHL status. The occupational population faced the same health problems as the general population and health threats from various complex occupational hazards in the working environment. Thus, a special scale was needed. The NHLMQ (2014 edition) was amended according to the expert advice and the traits of jobs among the participants to adopt the investigation for evaluating the OHL in this study (Supplementary material: Questionnaire 1). This scale of OHL consisted of four dimensions of occupational health knowledge (OHK), occupational health attitude (OHA), occupational health behavior (OHB), and occupational health skills (OHS), with a total of 64 items. It covered basic occupational health and safety knowledge involving personal protection, emergency measures, and hazard (or sign) recognition. If the participants answered the question correctly, they would get a point; otherwise, they would not get any points. The total score of OHL was 64, and the full scores of each dimension were 30 (OHK), 8 (OHA), 7 (OHB), and 19 (OHS). If the score of the participants reached 80% or above, then they had sufficient OHL. That is, the score was OHL ≥ 52. Similarly, if the scores of OHK, OHA, OHB, and OHK reached 24, 7, 6, and 16 or above, respectively, the participants had sufficient literacy in these dimensions [13]. The questionnaire was reliable in this study, with a Cronbach’s α confidence of 0.808 (95% confidence interval (CI): 0.788–0.827) [18]. Bartlett’s test of sphericity was significant, with p-value < 0.001 and Kaiser-Meyer-Olkin = 0.908, suggesting that the construct validity of this questionnaire was satisfactory. We further evaluated the criterion-related validity based on correlation and discriminant analysis. Although the correlation and discriminant analysis results were not highly satisfactory, we suggested that there were still correlations between the OHL and criteria (Supplementary material: Criterion-related validity evaluation, Table S1 and Figure S1).

### Variables and covariables

To estimate the association, the OHL and its four dimensions (OHK, OHA, OHB, and OHK) were considered the dependent variables, and length of service and job category were the independent variables. The need for a special note was based on occupational characteristics in the port. The job category had been divided into six categories: (1) longshoreman, (2) skilled worker (e.g., repairman, wireman, and welder), (3) driver, (4) inspector (e.g., inspection staff, tallyman, and receiving clerk), (5) security guard (e.g., security personnel, guard, and patrolman), and (6) others (e.g., manager, engineer, and clerk. Clear definitions are presented in Table S2. The covariables included age, sex (Female/Male), educational level (college degree or above/middle or high school/primary school or below), and marital status (No/Yes/Other) according to a directed acyclic graph (Figure S2).

### Statistical analysis

Continuous variables were expressed using mean and standard deviation, whereas categorical variables were described using the number of cases and composition ratio. T-test and chi-square test were performed to compare the differences of OHL (or the four dimensions) among the two groups according to variable type. Binary logistic regression was initially conducted to estimate the association between OHL (or the four dimensions) and interesting factors. For the binary logistic model, if individuals had sufficient OHL (or the four dimensions), the dependent variable would be mapped to 1; otherwise, the variable would be mapped to 0. Given the significance of dynamically assessing the effects of interesting factors on promoting OHL, the OHL, OHK, and OHS scores were stratified into quartiles (Q1–Q4), using the first quartile as a reference. In addition, ordinal logistic regression was conducted for further analysis. Moreover, the crud model without any covariates and the adjusted model were used. Given that the education level was an important confounding factor, we further executed a subgroup analysis stratified by education level. Meanwhile, two sensitivity analyses were also performed to analyze the association. The exclusion criteria were (1) those who took more than 90% of *Time*_*total*_ or less than 10% of *Time*_*total*_, including those with the *Time*_*total*_ range of 10–90% (12–45 min), and (2) those who took more than 99% of *Time*_*total*_ or less than the 1% of *Time*_*total*_, including those with the *Time*_*total*_ range of 1–99% (7–137 min). The analyses were conducted using R software (version 4.1.3). A p-value of < 0.05 was considered statistically significant.

## Result

### Characteristics of the participants

Table 1 shows the result of demographic characteristics and OHL status of the study population. The mean age of the participants was 40.2 years old (range: 18–60 years old). The majority of them were male (95.30%), attended middle or high schools (65.40%), married (74.90%), and longshoremen (26.00%). The mean length of service was 15.5 years. The result indicated that 72.90% of the participants had sufficient OHL, with a mean score (standard deviation) of 53.10 (7.26), and the proportions of those who had sufficient OHK, OHA, OHB, and OHS were 63.50%, 85.70%, 95.60%, and 56.60%, respectively.

**Table 1:** Basic information of participants

Characteristics		n (%) or Mean ± SD	
Sample size			3492
Age (years old)			40.2 (9.91)
Sex	Female		163 (4.70%)
	Male		3329 (95.30%)
Education	College degree or above		1048 (30.00%)
	Middle or high schools		2283 (65.40%)
	Primary school or below		161 (4.60%)
Marriage	No		744 (21.30%)
	Yes		2617 (74.90%)
	Other		131 (3.80%)
Length of service (years)			15.5 (9.65)
Job category	Longshoreman		907 (26.00%)
	Skilled worker		473 (13.50%)
	Driver		454 (13.00%)
	Inspector		236 (6.80%)
	Security guard		107 (3.10%)
	Other		1315 (37.70%)
Occupational Health Literacy	Score		53.10 (7.26)
	Yes		2547 (72.90%)
	No		945 (27.10%)
Occupational Health Knowledges	Score		23.60(4.86)
	Yes		2217 (63.50%)
	No		1275 (36.50%)
Occupational Health Attitude	Score		7.34 (1.40)
	Yes		2992 (85.70%)
	No		500 (14.30%)
Occupational Health Behaviors	Score		6.79 (0.76)
	Yes		3339 (95.60%)
	No		153 (4.40%)
Occupational Health Skills	Score		15.3 (2.02)
	Yes		1975 (56.60%)
	No		1517 (43.40%)

Footnotes: SD: standard deviation; Score threshold of participants who have sufficient occupational health literacy: score of Occupational Health Literacy ≥ 52; score of Occupational Health Knowledges ≥ 24; score of Occupational Health Attitude ≥ 7; score of Occupational Health Behaviors ≥ 6; score of Occupational Health Skills ≥ 16

### Distribution characteristics of OHL in the length of service and job category

Generally, the difference between OHL and the four dimensions in the length of service and job category was statistically significant. In comparison with those who lack sufficient OHL (or OHK, OHA, OHB, and OHS), the mean length of service and the proportion of the longer length of service were more likely to be longer and higher than those who have sufficient OHL (or OHK, OHA, OHB, and OHS). For the job category, compared with those who lack sufficient OHL (or OHK, OHA, OHB, and OHS), the proportion of skilled workers, inspectors, and security guards was higher, and the proportion of longshoremen and drivers was lower than those who have sufficient OHL (or OHK, OHA, OHB, and OHS; Table 2).

**Table 2:** Distribution characteristics of Occupational Health Literacy in length of service and job category

		Occupational Health Literacy		Occupational Health Knowledges		Occupational Health Attitude		Occupational Health Behaviors		Occupational Health Skills	
		Yes	No	Yes	No	Yes	Yes	No	Yes	No	Yes
Length of service (years)^@^		15.9 (9.42)	14.3 (10.2)	16.0 (9.42)	14.5 (9.96)	15.7 (9.59)	14.2 (9.94)	15.5 (9.65)	13.9 (9.53)	15.8 (9.47)	15.0 (9.87)
	*t-value*	4.117		-4.555		-3.035		-2.06		-2.471	
	*p*	< 0.001^*^		<0.001^*^		0.003^*^		0.041^*^		0.014^*^	
	≤ 32	620 (24.3%)	324 (34.3%)	530 (23.9%)	414 (32.5%)	777 (26.0%)	167 (33.4%)	893 (26.7%)	51 (33.3%)	495 (25.1%)	449 (29.6%)
	33–40	635 (24.9%)	214 (22.6%)	550 (24.8%)	299 (23.5%)	723 (24.2%)	126 (25.2%)	811 (24.3%)	38 (24.8%)	475 (24.1%)	374 (24.7%)
	41–49	638 (25.0%)	190 (20.1%)	558 (25.2%)	270 (21.2%)	732 (24.5%)	96 (19.2%)	795 (23.8%)	33 (21.6%)	497 (25.2%)	331 (21.8%)
	≥ 50	654 (25.7%)	217 (23.0%)	579 (26.1%)	292 (22.9%)	760 (25.4%)	111 (22.2%)	840 (25.2%)	31 (20.3%)	508 (25.7%)	363 (23.9%)
	*χ* ^*2*^	35.829		31.372		15.708		4.083		11.809	
	*p*	< 0.001*		< 0.001*		0.001*		0.253		0.008*	
Job category	Longshoreman	550 (21.6%)	357 (37.8%)	466 (21.0%)	441 (34.6%)	734 (24.5%)	173 (34.6%)	858 (25.7%)	49 (32.0%)	438 (22.2%)	469 (30.9%)
	Skilled worker	398 (15.6%)	75 (7.9%)	364 (16.4%)	109 (8.5%)	435 (14.5%)	38 (7.6%)	468 (14.0%)	5 (3.3%)	282 (14.3%)	191 (12.6%)
	Driver	299 (11.7%)	155 (16.4%)	254 (11.5%)	200 (15.7%)	363 (12.1%)	91 (18.2%)	420 (12.6%)	34 (22.2%)	237 (12.0%)	217 (14.3%)
	Inspector	180 (7.1%)	56 (5.9%)	156 (7.0%)	80 (6.3%)	206 (6.9%)	30 (6.0%)	224 (6.7%)	12 (7.8%)	150 (7.6%)	86 (5.7%)
	Security guard	81 (3.2%)	26 (2.8%)	64 (2.9%)	43 (3.4%)	93 (3.1%)	14 (2.8%)	104 (3.1%)	3 (2.0%)	69 (3.5%)	38 (2.5%)
	Other	1039 (40.8%)	276 (29.2%)	913 (41.2%)	402 (31.5%)	1161 (38.8%)	154 (30.8%)	1265 (37.9%)	50 (32.7%)	799 (40.5%)	516 (34.0%)
	*χ* ^*2*^	137.436		126.872		51.995		27.166		47.436	
	*p*	< 0.001*		< 0.001*		<0.001*		< 0.001*		< 0.001*	

Footnotes:* *P < 0.05* was considered statistically significant;^@^ The variable was continuous, and described by mean (standard deviation), other variables were categorical and described by number (proportion); Score threshold of participants who have sufficient occupational health literacy: score of Occupational Health Literacy ≥ 52; score of Occupational Health Knowledges ≥ 24; score of Occupational Health Attitude ≥ 7; score of Occupational Health Behaviors ≥ 6; score of Occupational Health Skills ≥ 16

### Association analysis based on binary logistic regression

The analysis indicated that the association between length of service or job category and OHL (or OHK, OHA, OHB, and OHS) was statistically significant (Table 3). For OHL, *Crude OR* increased with the length of service level, and this trend was more pronounced in the adjusted model. The *Adjusted OR* (95% CI) of the length of service increased from 1.11 (1.06–1.16) in the 33–40 years group to 1.19 (1.13–1.26) in ≥ 50 years group, and this growth trend was significant (*P*_*trend*_ <0.001). This result suggested that subjects with a longer length of service would be more likely to have sufficient OHL. Similar associations were observed for OHK, OHA, and OHB but not for OHB (Table 3 and S2).

**Table 3:** Relationship between Occupational Health Literacy and length of service or job category based on binary logistic regression

		Occupational Health Literacy *OR (95%CI)*	Occupational Health Knowledges *OR (95%CI)*	Occupational Health Attitude *OR (95%CI)*	Occupational Health Behaviors *OR (95%CI)*	Occupational Health Skills *OR (95%CI)*
Length of service (years, ref = Length of service with ≤ 32 years)						
33–40	*Crude*	1.10(1.05 ~ 1.14)*	1.09(1.04 ~ 1.14)*	1.03(1.00 ~ 1.06)	1.01(0.99 ~ 1.03)	1.04(0.99 ~ 1.08)
	*Adjusted*	1.11(1.06 ~ 1.16)*	1.10(1.06 ~ 1.16)*	1.04(1.00 ~ 1.07)*	1.01(0.99 ~ 1.03)	1.05(1.00 ~ 1.11)*
41–49	*Crude*	1.12(1.08 ~ 1.17)*	1.12(1.07 ~ 1.17)*	1.06(1.03 ~ 1.10)*	1.01(1.00 ~ 1.03)	1.08(1.03 ~ 1.13)*
	*Adjusted*	1.14(1.09 ~ 1.19)*	1.13(1.08 ~ 1.19)*	1.07(1.03 ~ 1.11)*	1.01(0.99 ~ 1.03)	1.10(1.05 ~ 1.16)*
≥ 50	*Crude*	1.10(1.05 ~ 1.14)*	1.11(1.06 ~ 1.16)*	1.05(1.02 ~ 1.09)*	1.02(1.00 ~ 1.04)	1.06(1.01 ~ 1.11)*
	*Adjusted*	1.19(1.13 ~ 1.26)*	1.20(1.14 ~ 1.27)*	1.09(1.05 ~ 1.14)*	1.02(0.99 ~ 1.04)	1.13(1.06 ~ 1.20)*
Job category (ref = Security guard)						
Longshoreman	*Crude*	0.86(0.79 ~ 0.94)*	0.92(0.84 ~ 1.01)	0.94(0.88 ~ 1.01)	0.97(0.94 ~ 1.02)	0.85(0.77 ~ 0.94)*
	*Adjusted*	0.90(0.83 ~ 0.98)*	0.96(0.88 ~ 1.06)	0.97(0.91 ~ 1.05)	0.97(0.93 ~ 1.01)	0.89(0.80 ~ 0.98)*
Skilled worker	*Crude*	1.09(0.99 ~ 1.19)	1.19(1.07 ~ 1.31)*	1.05(0.98 ~ 1.13)	1.02(0.97 ~ 1.06)	0.95(0.86 ~ 1.06)
	*Adjusted*	1.04(0.95 ~ 1.14)	1.11(1.01 ~ 1.23)*	1.04(0.97 ~ 1.12)	1.01(0.97 ~ 1.06)	0.95(0.85 ~ 1.05)
Driver	*Crude*	0.91(0.83 ~ 0.99)*	0.96(0.87 ~ 1.06)	0.93(0.87 ~ 1.00)	0.95(0.91 ~ 1.00)*	0.88(0.80 ~ 0.98)*
	*Adjusted*	0.91(0.83 ~ 0.99)*	0.96(0.87 ~ 1.06)	0.94(0.87 ~ 1.01)	0.95(0.91 ~ 1.00)*	0.89(0.80 ~ 0.99)*
Inspector	*Crude*	1.01(0.91 ~ 1.11)	1.06(0.96 ~ 1.19)	1.00(0.93 ~ 1.09)	0.98(0.93 ~ 1.02)	0.99(0.89 ~ 1.11)
	*Adjusted*	0.97(0.88 ~ 1.08)	1.03(0.92 ~ 1.14)	0.99(0.91 ~ 1.07)	0.98(0.93 ~ 1.02)	1.01(0.90 ~ 1.13)

Footnotes: *P < 0.05 was considered statistically significant; *OR*: odds rate; 95%CI: 95% confidence interval; *Crude*: no adjustment; *Adjusted*: adjusted for age, sex, education and marriage; ref: reference

For OHL, the association with skilled workers or inspectors was insignificant, but the association was significant with longshoremen or drivers, with *Crude OR* (95% CI) of 0.86 (0.79–0.94) and 0.91 (0.83–0.99), respectively. Remarkably, whether the association was significant, *Crude OR* and *Adjusted OR* was close to 1. Therefore, although the association between job category and OHL was significant, the effect was weak. The results were roughly the same for OHK, OHA, OHB, and OHS (Table 3 and S3).

### Association analysis based on ordinal logistic regression

Ordinal logistic regression was conducted to estimate the dynamic effects of length of service and job category on the improvement of OHL, OHK, and OHS. The results are shown in Table 4. For length of service, the ability of the 33–40 years group to improve their OHL was 1.5 times that of the ≤ 32 years group (*Adjusted OR* = 1.50, 95% CI: 1.25–1.79). This ability would improve with an increasing length of service (41–49 years group, *Adjusted OR* = 1.75; ≥50 years group, *Adjusted OR* = 2.19), which indicated that length of service could promote the improvement of OHL continuously. In addition, the same results were observed in OHK and OHS (Table 4 and S4).

**Table 4:** Relationship between Occupational Health Literacy and length of service or job category based on ordinal logistic regression

		Occupational Health Literacy *OR (95%CI)*	Occupational Health Knowledges *OR (95%CI)*	Occupational Health Skills *OR (95%CI)*
Length of service (years, ref = Length of service with ≤ 32 years)				
33–40	*Crude*	1.40(1.18 ~ 1.65)*	1.44(1.22 ~ 1.70)*	1.13(0.95 ~ 1.35)
	*Adjusted*	1.50(1.25 ~ 1.79)*	1.54(1.29 ~ 1.85)*	1.22(1.02 ~ 1.47)*
41–49	*Crude*	1.60(1.35 ~ 1.90)*	1.63(1.37 ~ 1.93)*	1.32(1.11 ~ 1.57)*
	*Adjusted*	1.75(1.44 ~ 2.14)*	1.77(1.45 ~ 2.16)*	1.49(1.22 ~ 1.83)*
≥ 50	*Crude*	1.48(1.25 ~ 1.75)*	1.45(1.23 ~ 1.71)*	1.23(1.04 ~ 1.46)*
	*Adjusted*	2.19(1.74 ~ 2.75)*	2.22(1.77 ~ 2.78)*	1.56(1.24 ~ 1.98)*
Job category (ref = Security guard)				
Longshoreman	*Crude*	0.69(0.49 ~ 0.99)*	0.68(0.48 ~ 0.97)*	0.58(0.40 ~ 0.83)*
	*Adjusted*	0.87(0.60 ~ 1.25)	0.87(0.60 ~ 1.25)	0.68(0.47 ~ 0.99)*
Skilled worker	*Crude*	1.91(1.32 ~ 2.76)*	1.83(1.26 ~ 2.64)*	0.85(0.58 ~ 1.24)
	*Adjusted*	1.60(1.09 ~ 2.34)*	1.45(0.99 ~ 2.12)	0.85(0.58 ~ 1.26)
Driver	*Crude*	0.79(0.54 ~ 1.14)	0.81(0.56 ~ 1.18)	0.66(0.45 ~ 0.96)*
	*Adjusted*	0.80(0.55 ~ 1.17)	0.82(0.56 ~ 1.20)	0.68(0.46 ~ 1.00)*
Inspector	*Crude*	1.33(0.89 ~ 1.99)	1.17(0.78 ~ 1.75)	0.97(0.64 ~ 1.46)
	*Adjusted*	1.20(0.80 ~ 1.81)	0.99(0.65 ~ 1.49)	1.06(0.70 ~ 1.61)

Footnotes: * *P < 0.05* was considered statistically significant; *OR*: odds rate; 95%CI: 95% confidence interval; *Crude*: no adjustment; *Adjusted*: adjusted for age, sex, education and marriage; ref: reference; The Occupational Health attitude and Occupational Health Behaviors indicators were not included, because their scores did not meet the requirements of quartile; All the ordinal logistic regression models passed the parallel test (*Probability > 0.05*, Table S4)

For job category, the ability was mostly not associated with the improvement of OHL in the adjusted model. However, notably, compared with security guards, the ability of skilled workers to improve OHL was 1.60 times that of the former (*Adjusted OR* = 1.60, 95% CI: 1.09–2.34), which indicated that this ability of skilled workers was better. By contrast, in the adjusted model, the ability of longshoremen (*Adjusted OR* = 0.68, 95% CI: 0.47–0.99) and drivers (*Adjusted OR* = 0.68, 95% CI: 0.46–1.00) to improve the OHS was 0.68 times that of the security guards, indicating that this ability of longshoremen and drivers was worse (Table 4 and S4).

### Subgroup analysis and sensitivity analysis results

**Fig. 2:**
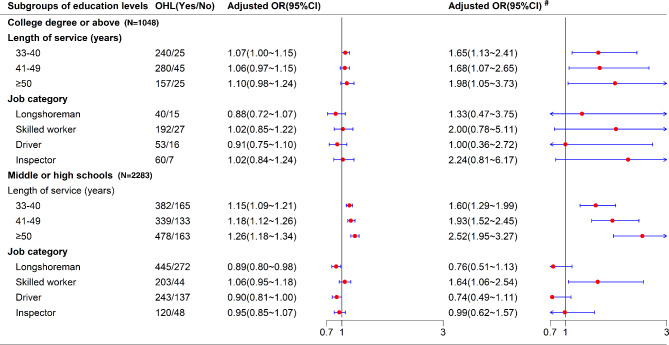
Relationship between occupational health literacy (OHL) and length of service or job category stratified by education levels. *OR*: odds rate; 95%CI: 95% confidence interval; *Crude*: no adjustment; *Adjusted*: adjusted for age, sex, education and marriage; ^#^ the *OR* was estimated by ordinal logistic regression. The education level of primary school or below was not stratified, because of the insufficient sample size

Subgroup analysis stratified by education levels were presented in Fig. 2 and Tables S5–S6; the results were consistent with the main finding above. In general, the association between length of service and OHL was statistically significant and showed an evident trend with an increase in the length of service, particularly in the subgroup of middle or high school education level. However, this association between job category and OHL was generally not significant. Therefore, these results should be cautiously interpreted because of the sample size limitation in those subgroups. The sensitivity analysis results were materially unchanged from those of the original results, after including the samples with the *Time*_*total*_ range of 10–90% or 1–99% (Tables S7–S12).

## Discussion

This cross-sectional study assessed the OHL status of port employees in Shenzhen and analyzed the association between length of service and job category with OHL and its four dimensions. The main findings were as follows. (1) Of the participants, 72.90% had sufficient OHL, with a mean score (standard deviation) of 53.10 (7.26). (2) The binary logistic regression indicated that the association between length of service and OHL was statistically significant and that *Adjusted OR* would increase with the length of service level. The association between longshoremen or drivers and OHL was also significant, but the effect was weak. (3) The ordinal logistic regression indicated that length of service could continuously promote OHL improvement. Moreover, most job categories were not associated with improving OHL, but the ability to improve the OHL of skilled workers was better.

Previous studies assessing and exploring the OHL status and its influencing factors in the occupational population are limited. Although some studies have focused on the occupational population, the evaluation index was HL, not OHL. Yang et al. surveyed 3507 employees from 13 enterprises in Hebei Province, China and found that the total OHL level was 32.24% [19]. Pak-Leng et al. utilized the Health Literacy Instrument to investigate Filipino domestic workers and found that only 37.4% of the respondents had sufficient HL [20]. Compared with these studies, the present work surveyed port employees using a special questionnaire. The finding indicated that 72.90% of the participants had sufficient OHL, which was a higher level. This result suggested that most participants could understand and apply basic OHK and OHS. This comparison seems unreasonable because of the different evaluation indexes (HL and OHL). However, in previous studies, the questionnaire score of 80% or more of the total score was considered as having sufficient HL (or OHL). On the basis of this criterion, the above comparison may be reasonable to some extent, but the comparison results should be interpreted with caution. The growing number of studies devoted to providing a clear definition of OHL and proposing corresponding evaluation programs may contribute to promoting the research of the OHL of employees in different countries, cultural backgrounds, and industries [5, 21].

As a personal health resource, the emphasis on HL, in addition to reducing the risk of occupational accidents and diseases [22], can affect the ability to work [22]. Therefore, the importance of OHL in occupational health promotion must be continuously emphasized. The study showed that with the length of service, the OHL level and the ability to improve OHL increased sharply, and *Adjusted OR* increased from 1.5 to 2.19 (in ordered logistic regression), which fully demonstrated the promoting effect of length of service on OHL. The employees with longer service years would have more opportunities for skill training and occupational health education during their careers. Institutional occupational protection actions have been continuously implemented. Companies commission occupational health institutions to conduct the following measures every year. These measures constitute a closed loop: monitoring, feedback, and action are the three key points in this closed loop. Detection of hazardous factors in the working environment, health management, and occupational disease monitoring, provide the based information and requirements (monitoring). Based on this information, occupational health and safety specialists provide feedback on employees’ health promotion programs, and finally, these plans will be implemented (action, Figure S3). Occupational health education and assessment play important roles in the link to activate. Thus, pre-job education and high frequency of occupational health training and regular assessment during the career had become the key to improving their OHL.

We developed specialized occupational health education methods to help employees acquire OHK, considering the differences in job categories and exposure to occupational hazards. For example, for job categories involving exposure to chemical toxins (such as longshoremen or inspectors), we provided education on the toxicological properties of chemicals, symptoms of poisoning, preventive measures, and organized emergency drills. For job categories that involve exposure to high temperatures (such as drivers or longshoremen), we required employees to acquire relevant knowledge and first aid skills for preventing heat stroke and emphasized the importance of electrolyte and water supplementation. For job categories that involve exposure to electromagnetic radiation (such as wiremen or welders), we provided training on personal protective measures and first aid skills for electric shock. All of the aforementioned intervening measures can explain the high level of the participants having sufficient OHL to a certain degree. Therefore, the effect of length of service on OHL can be indirectly understood as the cumulative dose effect of occupational health promotion. Conversely, occupational health education implements through occupational health protection [23]; thus, the positive association between OHL and length of service also tests the effect of occupational health protection.

Generally, different jobs require employees to have different occupational skills and background knowledge. Therefore, the OHL of people in different job categories should differ. Saowanee et al. and Ying et al. supported this conclusion [24, 25]. The difference is that, in this study, a significant association existed between some job categories and OHL. However, overall, the strength of this association was not apparent, which may be related to the ongoing occupational health education among port employees, as mentioned previously. An alternative explanation suggested that the emphasis of occupational health education varied from position to position, but some general and necessary OHK was repeatedly emphasized to ensure that employees in different jobs could understand this necessary knowledge. Furthermore, it was also related to the design of the questionnaire, which contains the basic knowledge of occupational health involving personal protection, emergency measures, and hazard (or sign) recognition. These knowledge and signs are often used or observed at work. Thus, they are relatively easy to grasp, which could explain the high level of the participants having sufficient OHL to a certain degree. Notably, ordered logistic regression analysis showed that skilled workers had a strong ability to improve OHL (*Adjusted OR* = 1.60), whereas longshoremen and drivers had a weak ability to improve OHS (*Adjusted OR* = 0.68), which may be related to their education level. Therefore, occupational health education must be strengthened for longshoremen and drivers.

Based on our experience, combining general occupational health education with specialized education tailored to different job positions is effective in improving the OHL of the port workforce. This approach ensures the dissemination of general knowledge about occupational health while increasing specific health skills for preventing occupational hazards (such as water and electrolyte supplementation for those exposed to high temperatures). As the first step in the overall occupational health promotion plan, dynamic risk monitoring and refined occupational health risk management will be two important intervention policies in the future, building on the continuous improvement of OHL. Examples are the dynamic monitoring of on-the-job blood pressure and heart rate for workers performing tasks at heights and the dynamic monitoring of peripheral blood glucose for employees with diabetes who work in hot environments. Furthermore, refined occupational health risk management aims to develop and utilize a health intelligence information system that is tailored to employees’ job categories. This system will enable real-time monitoring of employees’ health abnormalities and the timely dissemination of health management advice and other intervention measures to individuals and managers. This approach ensures that diseases are treated at the initial stage and helps to prevent accidents caused by unexpected health issues among employees effectively.

This study is not without limitations. First, the questionnaire applies only to port employees in Shenzhen; thus, the conclusion should be extrapolated cautiously. Second, the study’s sample size is small, and the statistical efficiency may be insufficient; thus, including more sample sizes is necessary. Third, the concept design and validity of the indicators, which were based on the concept of HL, for OHL were rarely discussed in the present study. Moreover, uniform norms and consensus for such study under existing conditions remained scarce, leading to a bad horizontal comparison and extension situation. Thus, further studies need to pay more attention to the above problems.

## Conclusion

This study showed that most port employees in Shenzhen had sufficient OHL. Moreover, length of service and job category could affect OHL, and the effect of length of service may be more obvious; that is, the length of service can promote the improvement of OHL continuously.

## Electronic supplementary material

Below is the link to the electronic supplementary material.

Supplementary Material 1

## Data Availability

The datasets used during the current study available from the corresponding author on reasonable request.

## List of Abbreviations

HL: Health literacy
NHLMQ: National Health Literacy Monitoring Questionnaire
OHL: Occupational health literacy
OHK: Occupational health knowledge
OHA: Occupational health attitude
OHB: Occupational health behavior
OHS: Occupational health skills
