# Supplementary material for: Association of length of service and job category with occupational health literacy of port employees in Shenzhen, China
Source: BMC Public Health. 2023 Jun 23;23:1223. doi: 10.1186/s12889-023-15769-7 (PMC10288714; doi:10.1186/s12889-023-15769-7)
Supplement: Supplementary file 1 — Supplementary Material 1 [file 12889_2023_15769_MOESM1_ESM.docx]

**Supplementary material**

**Association of Length of Service and Job Category with Occupational Health Literacy of Port Employees in Shenzhen, China**

**Sample size estimation**

The cross-sectional sampling survey sample size is calculated as follows:

$n=\left( Z_{\alpha}^{2}\times pq \right)/d^{2}$,

where *n* is the sample size, *Z* is the statistical quantity, *α* = 0.05, *Z* = 1.96, *p* is the expected prevalence, *q* = 1 − *p*, *d* is the allowable error, and *d* = 0.1*p.* Referencing to a previous study, the expected proportion of the respondents with sufficient HL was 22.3% [1]; thus, the minimum estimated sample size in theory was 683. Furthermore, the recommended rules of thumb of logistic regression analysis were event per variable of 50, and the formula is *n* = 100 + 50*i*, where *i* is the number of independent variables in the regression model [2]. Thus, the theoretical estimated sample size was 350. To summarize, the sample size of 3946 in this study was completely adequate.

**References:**

1. Li Z, Tian Y, Gong Z, Qian L: Health Literacy and Regional Heterogeneities in China: A Population-Based Study. *Frontiers in Public Health* 2021, 9.

2. Bujang MA, Sa'At N, Sidik T, Joo LC: Sample Size Guidelines for Logistic Regression from Observational Studies with Large Population: Emphasis on the Accuracy Between Statistics and Parameters Based on Real Life Clinical Data. *Malays J Med Sci* 2018, 25(4):122-130.

**Criterion-related validity evaluation**

Based on the assumption that sufficient occupational health literacy (OHL) can improve health conditions, we selected the health performance and outcomes including sport (Frequently/Occasionally), occupational stress (OS; Yes/No), hypertension (Yes/No), fasting blood glucose (Normal/Unnormal), triglyceride (Normal/Unnormal), total cholesterol (Normal/Unnormal), high-density lipoprotein cholesterol (HDL, Normal/Unnormal), and low-density lipoprotein cholesterol (LDL, Normal/Unnormal) as the criteria. All the criteria were gathered from the Occupational Health Survey for Port Employees project [1]. The information of sport habit was collected by a questionnaire and OS status was measured by the Effort-Reward Imbalance Questionnaire [2]. And other health outcomes were determined by the results of biochemical indicators. We calculated the Phi coefficient correlation [3], a measure of association for two binary variables, between OHL and health outcomes. Furthermore, we conducted a discriminant analysis using OHL as a predictor and estimated the area under the curve (AUC) based on logistic regression.

Results indicated that the correlations between OHL and criterions ranged from −0.001 to 0.126 (Table S1) and the AUC values in the discriminant analysis ranged from 0.47 to 0.75 (Figure S1). Although the correlation and discriminant analysis results were not highly satisfactory, we suggest correlations between the OHL and criterions, especially concerning OS, HDL, and LDL.

Table S1 Correlations between occupational health literacy and criterions

| Items | Sport | OS | Hypertension | FBG | TG | TC | HDL | LDL |
| --- | --- | --- | --- | --- | --- | --- | --- | --- |
| Phi coefficient | -0.013 | 0.126 | -0.017 | -0.001 | -0.009 | -0.010 | -0.112 | 0.055 |
| AUC | 0.51 | 0.67 | 0.48 | 0.49 | 0.51 | 0.47 | 0.75 | 0.61 |


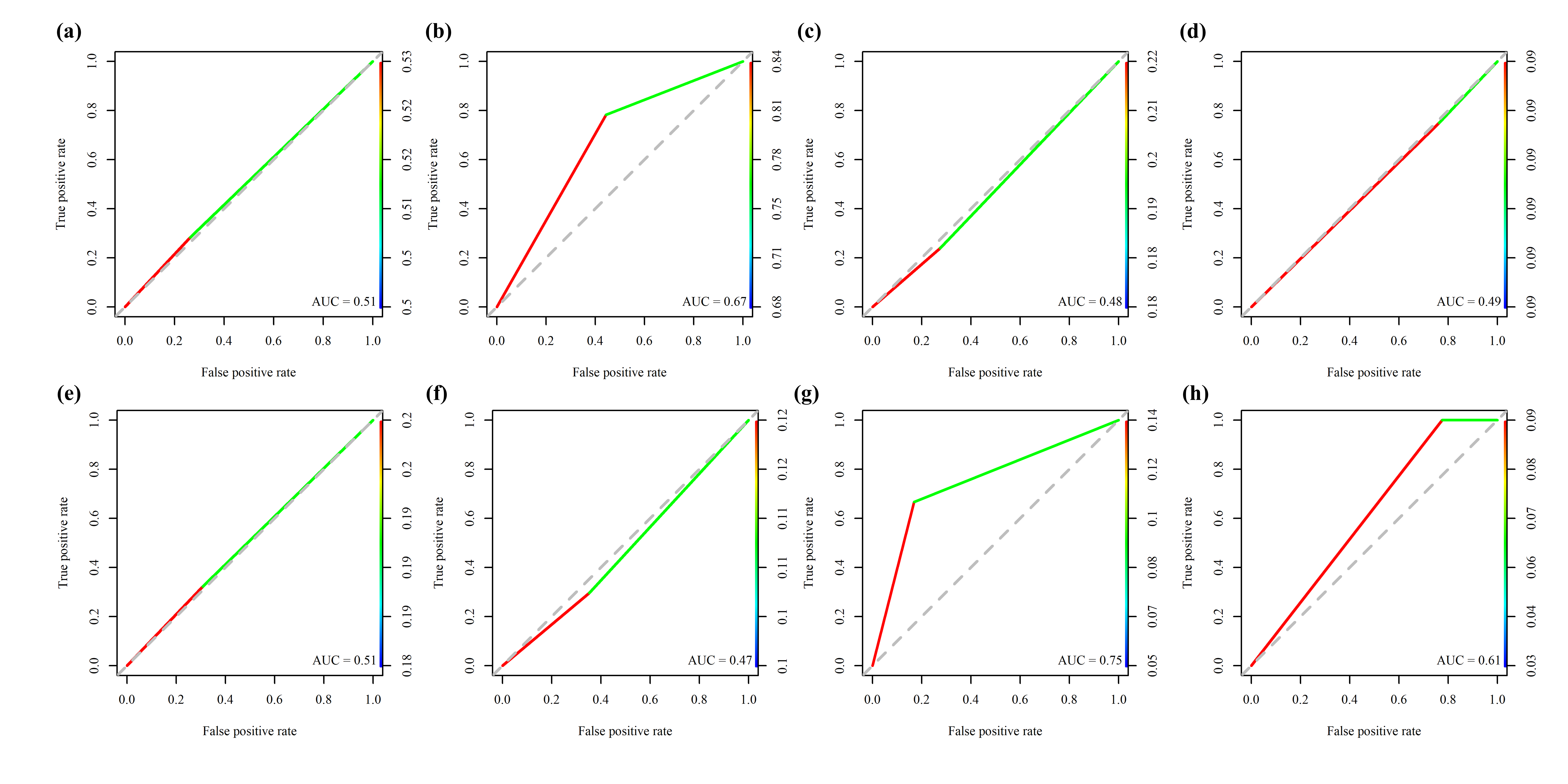


Figure S1 The predictive property by logistic regression using occupational health literacy as a predictor. a - h: discriminant variables were sport (Frequently/Occasionally), OS (Yes/No), hypertension (Yes/No), FBG(Normal/Unnormal), TG (Normal/Unnormal), TC (Normal/Unnormal), HDL (Normal/Unnormal), LDL (Normal/Unnormal), respectively.

**References:**

1. Wang J, Mo C, Huang Y, Lin D, Situ J, Zhang M, Zhang N: An Occupational Health Survey for Port Employees in Shenzhen and A Dataset Management System based on R. *medRxiv* 2022:2022-2026.

2. Bardhan R, Heaton K, Davis M, Chen P, Dickinson DA, Lungu CT: A Cross Sectional Study Evaluating Psychosocial Job Stress and Health Risk in Emergency Department Nurses. *Int J Environ Res Public Health* 2019, 16(18).

3. Cramer H: *Mathematical Methods of Statistics. Princeton: Princeton University Press*; 1946.

**Directed acyclic graph**

Length of service

Job category

Occupational health literacy

Demographic characteristics (age, sex, education and marriage)

Figure S2. The directed acyclic graph for all variables (abbreviated version)

**Tables**

Table S2 Definition for each job categorie

| Job category | Definition |
| --- | --- |
| Longshoreman | A person employed in a port to load and unload ships. The number of employees for this job accounted for the largest proportion in a port of Shenzhen. |
| Skilled worker | A Worker who has acquired special skills, including repairman, wireman, welder and plumber. |
| Driver | A person who drives a vehicle, loader, crane, forklift, excavator and tugboat. |
| Inspector | A person who inspects personnel, goods and vehicles, including inspection staff, tallyman and receiving clerk. |
| Security guard | A person employed to protect a building against intruders or damage, including security personnel, guard, and patrolman. |
| Other | Other jobs with a small number of employees, including manager, engineer, clerk, accountant, telephone operator, sailor, handyman, gas station attendants, and registrar. |

Table S3. Relationship between Occupational Health Literacy and length of service or job category based on binary logistic regression

|  |  | Occupational Health Literacy | | Occupational Health Knowledges | | Occupational Health Attitude | | Occupational Health Behaviors | | Occupational Health Skills | |
| --- | --- | --- | --- | --- | --- | --- | --- | --- | --- | --- | --- |
|  |  | *OR（95%CI）* | *P* | *OR（95%CI）* | *P* | *OR（95%CI）* | *P* | *OR（95%CI）* | *P* | *OR（95%CI）* | *P* |
| Length of service (years, ref= Length of service with ≤32 years) | | | | |  |  |  |  |  |  |  |
| 33-40 | *Crude* | 1.10(1.05~1.14)* | <0.01 | 1.09(1.04~1.14)* | <0.01 | 1.03(1.00~1.06) | 0.08 | 1.01(0.99~1.03) | 0.34 | 1.04(0.99~1.08) | 0.13 |
|  | *Adjusted* | 1.11(1.06~1.16)* | <0.01 | 1.10(1.06~1.16)* | <0.01 | 1.04(1.00~1.07)* | 0.05 | 1.01(0.99~1.03) | 0.44 | 1.05(1.00~1.11)* | 0.03 |
| 41-49 | *Crude* | 1.12(1.08~1.17)* | <0.01 | 1.12(1.07~1.17)* | <0.01 | 1.06(1.03~1.10)* | <0.01 | 1.01(1.00~1.03) | 0.15 | 1.08(1.03~1.13)* | <0.01 |
|  | *Adjusted* | 1.14(1.09~1.19)* | <0.01 | 1.13(1.08~1.19)* | <0.01 | 1.07(1.03~1.11)* | <0.01 | 1.01(0.99~1.03) | 0.31 | 1.10(1.05~1.16)* | <0.01 |
| ≥50 | *Crude* | 1.10(1.05~1.14)* | <0.01 | 1.11(1.06~1.16)* | <0.01 | 1.05(1.02~1.09)* | <0.01 | 1.02(1.00~1.04) | 0.06 | 1.06(1.01~1.11)* | 0.01 |
|  | *Adjusted* | 1.19(1.13~1.26)* | <0.01 | 1.20(1.14~1.27)* | <0.01 | 1.09(1.05~1.14)* | <0.01 | 1.02(0.99~1.04) | 0.18 | 1.13(1.06~1.20)* | 0.00 |
| Job category (ref= Security guard) | | | |  |  |  |  |  |  |  |  |
| Longshoreman | *Crude* | 0.86(0.79~0.94)* | <0.01 | 0.92(0.84~1.01) | 0.08 | 0.94(0.88~1.01) | 0.09 | 0.97(0.94~1.02) | 0.21 | 0.85(0.77~0.94)* | <0.01 |
|  | *Adjusted* | 0.90(0.83~0.98)* | 0.02 | 0.96(0.88~1.06) | 0.45 | 0.97(0.91~1.05) | 0.45 | 0.97(0.93~1.01) | 0.17 | 0.89(0.80~0.98)* | 0.02 |
| Skilled worker | *Crude* | 1.09(0.99~1.19) | 0.07 | 1.19(1.07~1.31)* | <0.01 | 1.05(0.98~1.13) | 0.18 | 1.02(0.97~1.06) | 0.42 | 0.95(0.86~1.06) | 0.36 |
|  | *Adjusted* | 1.04(0.95~1.14) | 0.42 | 1.11(1.01~1.23)* | 0.03 | 1.04(0.97~1.12) | 0.29 | 1.01(0.97~1.06) | 0.58 | 0.95(0.85~1.05) | 0.31 |
| Driver | *Crude* | 0.91(0.83~0.99)* | 0.04 | 0.96(0.87~1.06) | 0.45 | 0.93(0.87~1.00) | 0.06 | 0.95(0.91~1.00)* | 0.03 | 0.88(0.80~0.98)* | 0.02 |
|  | *Adjusted* | 0.91(0.83~0.99)* | 0.04 | 0.96(0.87~1.06) | 0.46 | 0.94(0.87~1.01) | 0.08 | 0.95(0.91~1.00)* | 0.03 | 0.89(0.80~0.99)* | 0.03 |
| Inspector | *Crude* | 1.01(0.91~1.11) | 0.91 | 1.06(0.96~1.19) | 0.25 | 1.00(0.93~1.09) | 0.93 | 0.98(0.93~1.02) | 0.34 | 0.99(0.89~1.11) | 0.87 |
|  | *Adjusted* | 0.97(0.88~1.08) | 0.62 | 1.03(0.92~1.14) | 0.64 | 0.99(0.91~1.07) | 0.75 | 0.98(0.93~1.02) | 0.31 | 1.01(0.90~1.13) | 0.83 |
| Other | *Crude* | 1.03(0.95~1.13) | 0.45 | 1.10(1.00~1.21)* | 0.04 | 1.01(0.95~1.09) | 0.69 | 0.99(0.95~1.03) | 0.63 | 0.96(0.87~1.06) | 0.45 |
|  | *Adjusted* | 0.98(0.90~1.07) | 0.61 | 1.03(0.93~1.13) | 0.60 | 1.00(0.93~1.07) | 0.91 | 0.99(0.95~1.03) | 0.50 | 0.96(0.87~1.06) | 0.45 |

Footnotes: **P<0.05* was considered statistically significant; *OR*: odds rate; *95%CI*: 95% confidence interval; *Crude:* no adjustment; *Adjusted*: adjusted for age, sex, education and marriage; ref: reference.

Table S4. Relationship between Occupational Health Literacy and the length of service or job category based on ordinal logistic regression

|  |  | Occupational Health Literacy | | | Occupational Health Knowledges | | | Occupational Health Skills | | |
| --- | --- | --- | --- | --- | --- | --- | --- | --- | --- | --- |
|  |  | *OR（95%CI）* | *P* | *Probability* | *OR（95%CI）* | *P* | *Probability* | *OR（95%CI）* | *P* | *Probability* |
| Length of service (years, ref= Length of service with ≤32 years) | | | |  |  |  |  |  |  |  |
| 33-40 | *Crude* | 1.40(1.18~1.65)* | <0.01 | 0.20 | 1.44(1.22~1.70)* | <0.01 | 0.55 | 1.13(0.95~1.35) | 0.15 | 0.90 |
|  | *Adjusted* | 1.50(1.25~1.79)* | <0.01 | 0.25 | 1.54(1.29~1.85)* | <0.01 | 0.69 | 1.22(1.02~1.47)* | 0.03 | 0.88 |
| 41-49 | *Crude* | 1.60(1.35~1.90)* | <0.01 | 0.29 | 1.63(1.37~1.93)* | <0.01 | 0.73 | 1.32(1.11~1.57)* | <0.01 | 0.16 |
|  | *Adjusted* | 1.75(1.44~2.14)* | <0.01 | 0.28 | 1.77(1.45~2.16)* | <0.01 | 0.91 | 1.49(1.22~1.83)* | <0.01 | 0.39 |
| ≥50 | *Crude* | 1.48(1.25~1.75)* | <0.01 | 0.49 | 1.45(1.23~1.71)* | <0.01 | 0.07 | 1.23(1.04~1.46)* | 0.02 | 0.73 |
|  | *Adjusted* | 2.19(1.74~2.75)* | <0.01 | 0.50 | 2.22(1.77~2.78)* | <0.01 | 0.55 | 1.56(1.24~1.98)* | <0.01 | 0.51 |
| Job category (ref= Security guard) | | |  |  |  |  |  |  |  |  |
| Longshoreman | *Crude* | 0.69(0.49~0.99)* | 0.04 | 0.05 | 0.68(0.48~0.97)* | 0.03 | 0.17 | 0.58(0.40~0.83)* | <0.01 | 0.31 |
|  | *Adjusted* | 0.87(0.60~1.25) | 0.44 | 0.05 | 0.87(0.60~1.25) | 0.44 | 0.13 | 0.68(0.47~0.99)* | 0.05 | 0.35 |
| Skilled worker | *Crude* | 1.91(1.32~2.76)* | <0.01 | 0.43 | 1.83(1.26~2.64)* | <0.01 | 0.42 | 0.85(0.58~1.24) | 0.40 | 0.43 |
|  | *Adjusted* | 1.60(1.09~2.34)* | 0.02 | 0.25 | 1.45(0.99~2.12) | 0.06 | 0.47 | 0.85(0.58~1.26) | 0.43 | 0.42 |
| Driver | *Crude* | 0.79(0.54~1.14) | 0.20 | 0.21 | 0.81(0.56~1.18) | 0.27 | 0.70 | 0.66(0.45~0.96)* | 0.03 | 0.71 |
|  | *Adjusted* | 0.80(0.55~1.17) | 0.25 | 0.16 | 0.82(0.56~1.20) | 0.30 | 0.65 | 0.68(0.46~1.00)* | 0.05 | 0.65 |
| Inspector | *Crude* | 1.33(0.89~1.99) | 0.17 | 0.30 | 1.17(0.78~1.75) | 0.45 | 0.52 | 0.97(0.64~1.46) | 0.87 | 0.59 |
|  | *Adjusted* | 1.20(0.80~1.81) | 0.38 | 0.14 | 0.99(0.65~1.49) | 0.95 | 0.31 | 1.06(0.70~1.61) | 0.79 | 0.60 |
| Other | *Crude* | 1.52(1.08~2.15)* | 0.02 | 0.26 | 1.55(1.10~2.19)* | 0.01 | 0.21 | 0.92(0.65~1.32) | 0.66 | 0.67 |
|  | *Adjusted* | 1.20(0.84~1.71) | 0.32 | 0.11 | 1.15(0.81~1.65) | 0.43 | 0.15 | 0.94(0.66~1.36) | 0.76 | 0.55 |

Footnotes: * *P<0.05* was considered statistically significant; *OR*: odds rate; *95%CI*: 95% confidence interval; *Crude:* no adjustment; *Adjusted*: adjusted for age, sex, education and marriage; ref: reference; The Occupational Health attitude and Occupational Health Behaviors indicators were not included, because their scores did not meet the requirements of quartile; All the ordinal logistic regression models passed the parallel test (*Probability >0.05*).

Table S5. Relationship between four dimensions of OHL and length of service or job category based on binary logistic regression stratified by education levels

| Subgroups of education levels | Occupational Health Knowledges | | Occupational Health Attitude | | Occupational Health Behaviors | | Occupational Health Skills | |
| --- | --- | --- | --- | --- | --- | --- | --- | --- |
|  | *Adjusted OR (95%CI)* | *P value* | *Adjusted OR (95%CI)* | *P value* | *Adjusted OR (95%CI)* | *P value* | *Adjusted OR (95%CI)* | *P value* |
| College degree or above (n=1048) |  |  |  |  |  |  |  |  |
| Length of service (years, ref= Length of service with ≤32 years) | | | |  |  |  |  |  |
| 33-40 | 1.11(1.02~1.21)* | 0.02 | 1.05(0.99~1.11) | 0.12 | 1.03(0.99~1.07) | 0.15 | 1.06(0.95~1.17) | 0.30 |
| 41-49 | 1.10(0.99~1.21) | 0.07 | 1.06(0.99~1.14) | 0.11 | 1.02(0.97~1.06) | 0.51 | 1.07(0.94~1.21) | 0.30 |
| ≥50 | 1.14(0.99~1.31) | 0.06 | 1.16(1.05~1.28)* | <0.01 | 1.04(0.97~1.10) | 0.29 | 1.06(0.90~1.26) | 0.47 |
| Job category (ref= Security guard) | | |  |  |  |  |  |  |
| Longshoreman | 0.97(0.77~1.23) | 0.83 | 1.10(0.93~1.30) | 0.28 | 0.96(0.86~1.07) | 0.45 | 0.83(0.63~1.11) | 0.21 |
| Skilled worker | 1.11(0.89~1.37) | 0.36 | 1.23(1.05~1.43)* | 0.01 | 0.99(0.89~1.09) | 0.80 | 0.80(0.62~1.04) | 0.10 |
| Driver | 0.93(0.74~1.17) | 0.52 | 1.13(0.96~1.34) | 0.13 | 0.93(0.83~1.03) | 0.15 | 0.82(0.62~1.09) | 0.17 |
| Inspector | 1.16(0.92~1.46) | 0.20 | 1.21(1.03~1.43)* | 0.02 | 0.92(0.83~1.02) | 0.11 | 0.99(0.75~1.31) | 0.94 |
| Other | 1.10(0.89~1.36) | 0.37 | 1.21(1.04~1.41)* | 0.02 | 0.96(0.87~1.06) | 0.39 | 0.86(0.67~1.12) | 0.27 |
| Middle or high schools (n=2283) |  |  |  |  |  |  |  |  |
| Length of service (years, ref= Length of service with ≤32 years) | | | |  |  |  |  |  |
| 33-40 | 1.12(1.06~1.18)* | <0.01 | 1.04(1.00~1.08) | 0.08 | 1.00(0.97~1.02) | 0.87 | 1.08(1.02~1.14)* | 0.01 |
| 41-49 | 1.16(1.09~1.24)* | <0.01 | 1.09(1.04~1.15)* | <0.01 | 1.01(0.98~1.03) | 0.71 | 1.14(1.07~1.22)* | <0.01 |
| ≥50 | 1.25(1.17~1.34)* | <0.01 | 1.10(1.04~1.15)* | <0.01 | 1.01(0.98~1.04) | 0.64 | 1.17(1.09~1.26)* | <0.01 |
| Job category (ref= Security guard) | |  |  |  |  |  |  |  |
| Longshoreman | 0.94(0.84~1.05) | 0.28 | 0.96(0.88~1.04) | 0.27 | 0.97(0.93~1.02) | 0.30 | 0.89(0.80~0.99)* | 0.04 |
| Skilled worker | 1.14(1.01~1.28)* | 0.04 | 1.02(0.94~1.12) | 0.60 | 1.02(0.97~1.07) | 0.52 | 0.99(0.88~1.12) | 0.87 |
| Driver | 0.96(0.86~1.08) | 0.49 | 0.91(0.83~0.98)* | 0.02 | 0.96(0.91~1.00) | 0.08 | 0.89(0.80~1.00)* | 0.05 |
| Inspector | 0.98(0.86~1.11) | 0.71 | 0.96(0.87~1.05) | 0.39 | 1.00(0.94~1.05) | 0.89 | 0.98(0.86~1.11) | 0.75 |
| Other | 0.99(0.88~1.10) | 0.77 | 0.96(0.89~1.04) | 0.32 | 0.99(0.95~1.04) | 0.72 | 0.96(0.86~1.07) | 0.50 |

Footnotes: OHL: Occupational health literacy; * *P<0.05* was considered statistically significant; *OR*: odds rate; *95%CI*: 95% confidence interval; *Crude:* no adjustment; *Adjusted*: adjusted for age, sex, education and marriage; ref: reference; The education level of primary school or below was not stratified, because of the insufficient sample size.

Table S6. Relationship between two dimensions of OHL and length of service or job category based on ordinal logistic regression stratified by education levels

| Subgroups of education levels | Occupational Health Knowledges | | Occupational Health Skills | |
| --- | --- | --- | --- | --- |
|  | *Adjusted OR (95%CI)* | *P value* | *Adjusted OR (95%CI)* | *P value* |
| College degree or above (n=1048) |  |  |  |  |
| Length of service (years, ref= Length of service with ≤32 years) | |  |  |  |
| 33-40 | 1.65(1.12~2.42)* | 0.01 | 1.30(0.88~1.92) | 0.18 |
| 41-49 | 1.56(0.98~2.48) | 0.06 | 1.45(0.90~2.32) | 0.13 |
| ≥50 | 1.40(0.73~2.65) | 0.31 | 1.46(0.75~2.85) | 0.26 |
| Job category (ref= Security guard) |  |  |  |  |
| Longshoreman | 0.95(0.34~2.66) | 0.92 | 0.57(0.20~1.63) | 0.29 |
| Skilled worker | 1.36(0.53~3.49) | 0.52 | 0.49(0.19~1.29) | 0.15 |
| Driver | 0.71(0.26~1.95) | 0.51 | 0.49(0.18~1.37) | 0.17 |
| Inspector | 1.12(0.41~3.07) | 0.82 | 0.91(0.32~2.53) | 0.86 |
| Other | 1.53(0.60~3.86) | 0.36 | 0.65(0.25~1.66) | 0.36 |
| Middle or high schools (n=2283) |  |  |  |  |
| Length of service (years, ref= Length of service with ≤32 years) | |  |  |  |
| 33-40 | 1.67(1.34~2.07)* | <0.01 | 1.30(1.04~1.62)* | 0.02 |
| 41-49 | 1.95(1.54~2.48)* | <0.01 | 1.61(1.26~2.06)* | <0.01 |
| ≥50 | 2.81(2.17~3.65)* | <0.01 | 1.71(1.31~2.24)* | <0.01 |
| Job category (ref= Security guard) |  |  |  |  |
| Longshoreman | 0.78(0.53~1.17) | 0.23 | 0.72(0.48~1.08) | 0.11 |
| Skilled worker | 1.52(0.98~2.36) | 0.06 | 0.99(0.63~1.55) | 0.96 |
| Driver | 0.80(0.53~1.20) | 0.28 | 0.70(0.46~1.06) | 0.09 |
| Inspector | 0.90(0.57~1.43) | 0.65 | 0.99(0.62~1.58) | 0.96 |
| Other | 0.95(0.64~1.4) | 0.78 | 0.96(0.64~1.43) | 0.83 |

Footnotes: OHL: Occupational health literacy; * *P<0.05* was considered statistically significant; *OR*: odds rate; *95%CI*: 95% confidence interval; *Crude:* no adjustment; *Adjusted*: adjusted for age, sex, education and marriage; ref: reference; The Occupational Health attitude and Occupational Health Behaviors indicators were not included, because their scores did not meet the requirements of quartile.

Table S7. Basic information of participants

(Sensitivity analysis in the population with *Time_total_* range of 10%~90%)

| Characteristics |  | n (%) or Mean ± SD | |
| --- | --- | --- | --- |
| Sample size |  | | 3041 |
| Age (years old) |  | | 40.3 (9.66) |
| Sex | Female | | 145 (4.8%) |
|  | Male | | 2896 (95.2%) |
| Education | College degree or above | | 887 (29.2%) |
|  | Middle or high schools | | 2022 (66.5%) |
|  | Primary school or below | | 132 (4.3%) |
| Marriage | No | | 117 (3.8%) |
|  | Yes | | 2315 (76.1%) |
|  | Other | | 609 (20.0%) |
| Length of service (years) |  | | 15.7 (9.50) |
| Job category | Longshoreman | | 784 (25.8%) |
|  | Skilled worker | | 397 (13.1%) |
|  | Driver | | 397 (13.1%) |
|  | Inspector | | 215 (7.1%) |
|  | Security guard | | 97 (3.2%) |
|  | Other | | 1151 (37.8%) |
| Occupational Health Literacy | Score | | 53.1 (7.28) |
|  | Yes | | 2211 (72.7%) |
|  | No | | 830 (27.3%) |
| Occupational Health Knowledges | Score | | 23.6 (4.88) |
|  | Yes | | 1922 (63.2%) |
|  | No | | 1119 (36.8%) |
| Occupational Health Attitude | Score | | 7.34 (1.42) |
|  | Yes | | 2608 (85.8%) |
|  | No | | 433 (14.2%) |
| Occupational Health Behaviors | Score | | 6.79 (0.748) |
|  | Yes | | 2908 (95.6%) |
|  | No | | 133 (4.4%) |
| Occupational Health Skills | Score | | 15.3 (2.01) |
|  | Yes | | 1730 (56.9%) |
|  | No | | 1311 (43.1%) |

Footnotes: *Time_total_*_:_ answer time of the total population; Score threshold of participants who have sufficient occupational health literacy: score of Occupational Health Literacy ≥ 52; score of Occupational Health Knowledges ≥ 24; score of Occupational Health Attitude ≥ 7; score of Occupational Health Behaviors ≥ 6; score of Occupational Health Skills ≥ 16.

Table S8. Relationship between Occupational Health Literacy and length of service or job category based on binary logistic regression

(Sensitivity analysis in the population with *Time_total_* range of 10%~90%)

|  |  | Occupational Health Literacy | | Occupational Health Knowledges | | Occupational Health Attitude | | Occupational Health Behaviors | | Occupational Health Skills | |
| --- | --- | --- | --- | --- | --- | --- | --- | --- | --- | --- | --- |
|  |  | *OR（95%CI）* | *P* | *OR（95%CI）* | *P* | *OR（95%CI）* | *P* | *OR（95%CI）* | *P* | *OR（95%CI）* | *P* |
| Length of service (years, ref= Length of service with ≤32 years) | | | | |  |  |  |  |  |  |  |
| 33-40 | *Crude* | 1.10(1.05~1.14)* | <0.01 | 1.09(1.04~1.13)* | <0.01 | 1.04(1.01~1.07)* | 0.02 | 1.01(0.99~1.03) | 0.37 | 1.04(1.00~1.10) | 0.08 |
|  | *Adjusted* | 1.12(1.07~1.17)* | <0.01 | 1.11(1.06~1.16)* | <0.01 | 1.04(1.00~1.08)* | 0.03 | 1.01(0.99~1.03) | 0.37 | 1.06(1.01~1.12)* | 0.02 |
| 41-49 | *Crude* | 1.11(1.07~1.16)* | <0.01 | 1.11(1.06~1.16)* | <0.01 | 1.06(1.03~1.10)* | <0.01 | 1.02(1.00~1.04) | 0.16 | 1.08(1.03~1.14)* | <0.01 |
|  | *Adjusted* | 1.15(1.09~1.20)* | <0.01 | 1.14(1.08~1.20)* | <0.01 | 1.08(1.04~1.12)* | <0.01 | 1.01(0.99~1.04) | 0.21 | 1.11(1.05~1.18)* | <0.01 |
| ≥50 | *Crude* | 1.09(1.05~1.13)* | <0.01 | 1.10(1.05~1.15)* | <0.01 | 1.06(1.02~1.09)* | <0.01 | 1.02(1.00~1.04) | 0.03 | 1.06(1.01~1.11)* | 0.02 |
|  | *Adjusted* | 1.20(1.14~1.27)* | <0.01 | 1.20(1.13~1.27)* | <0.01 | 1.10(1.05~1.15)* | <0.01 | 1.02(0.99~1.05) | 0.17 | 1.12(1.05~1.20)* | <0.01 |
| Job category (ref= Security guard) | | |  |  |  |  |  |  |  |  |  |
| Longshoreman | *Crude* | 0.87(0.8~0.95)* | 0.00 | 0.92(0.83~1.01) | 0.08 | 0.95(0.88~1.02) | 0.13 | 0.97(0.93~1.02) | 0.24 | 0.86(0.78~0.96)* | <0.01 |
|  | *Adjusted* | 0.92(0.84~1.01) | 0.07 | 0.96(0.87~1.06) | 0.39 | 0.98(0.91~1.06) | 0.61 | 0.97(0.93~1.02) | 0.22 | 0.90(0.81~1.00)* | 0.05 |
| Skilled worker | *Crude* | 1.10(1.00~1.20) | 0.06 | 1.17(1.05~1.3)* | <0.01 | 1.06(0.98~1.14) | 0.15 | 1.02(0.97~1.06) | 0.43 | 0.96(0.87~1.07) | 0.49 |
|  | *Adjusted* | 1.05(0.95~1.15) | 0.33 | 1.10(0.99~1.22) | 0.07 | 1.05(0.97~1.13) | 0.24 | 1.01(0.97~1.06) | 0.55 | 0.96(0.86~1.07) | 0.46 |
| Driver | *Crude* | 0.92(0.84~1.02) | 0.10 | 0.96(0.87~1.07) | 0.46 | 0.95(0.88~1.02) | 0.15 | 0.96(0.92~1.01) | 0.10 | 0.90(0.81~1.00) | 0.06 |
|  | *Adjusted* | 0.93(0.85~1.02) | 0.12 | 0.96(0.87~1.07) | 0.48 | 0.95(0.88~1.02) | 0.18 | 0.96(0.92~1.01) | 0.10 | 0.91(0.82~1.01) | 0.09 |
| Inspector | *Crude* | 1.02(0.92~1.13) | 0.72 | 1.05(0.94~1.17) | 0.41 | 1.01(0.93~1.10) | 0.80 | 0.98(0.93~1.02) | 0.34 | 1.00(0.89~1.12) | 0.99 |
|  | *Adjusted* | 0.99(0.89~1.10) | 0.85 | 1.01(0.90~1.13) | 0.86 | 1.00(0.92~1.08) | 0.92 | 0.98(0.93~1.02) | 0.32 | 1.03(0.91~1.15) | 0.67 |
| Other | *Crude* | 1.05(0.96~1.14) | 0.30 | 1.09(0.99~1.20) | 0.07 | 1.02(0.95~1.10) | 0.51 | 0.99(0.95~1.03) | 0.68 | 0.97(0.88~1.07) | 0.56 |
|  | *Adjusted* | 0.99(0.91~1.08) | 0.84 | 1.01(0.92~1.12) | 0.77 | 1.01(0.94~1.08) | 0.85 | 0.99(0.95~1.03) | 0.58 | 0.97(0.88~1.08) | 0.59 |

Footnotes: *Time_total_*_:_ answer time of the total population; **P<0.05* was considered statistically significant; *OR*: odds rate; *95%CI*: 95% confidence interval; *Crude:* no adjustment; *Adjusted*: adjusted for age, sex, education and marriage; ref: reference.

Table S9. Relationship between Occupational Health Literacy and the length of service or job category based on ordinal logistic regression

(Sensitivity analysis in the population with *Time_total_* range of 10%~90%)

|  |  | Occupational Health Literacy | | | Occupational Health Knowledges | | | Occupational Health Skills | | |
| --- | --- | --- | --- | --- | --- | --- | --- | --- | --- | --- |
|  |  | *OR（95%CI）* | *P* | *Probability* | *OR（95%CI）* | *P* | *Probability* | *OR（95%CI）* | *P* | *Probability* |
| Length of service (years, ref= Length of service with ≤32 years) | | | |  |  |  |  |  |  |  |
| 33-40 | *Crude* | 1.48(1.24~1.77)* | <0.01 | 0.17 | 1.51(1.27~1.80)* | <0.01 | 0.49 | 1.17(0.98~1.40) | 0.83 | 0.09 |
|  | *Adjusted* | 1.56(1.30~1.89)* | <0.01 | 0.20 | 1.58(1.31~1.91)* | <0.01 | 0.65 | 1.26(1.04~1.53)* | 0.83 | 0.02 |
| 41-49 | *Crude* | 1.66(1.39~1.98)* | <0.01 | 0.23 | 1.68(1.41~2.01)* | <0.01 | 0.44 | 1.33(1.11~1.59)* | 0.13 | 0.00 |
|  | *Adjusted* | 1.81(1.47~2.22)* | <0.01 | 0.20 | 1.81(1.47~2.23)* | <0.01 | 0.65 | 1.51(1.22~1.87)* | 0.24 | 0.00 |
| ≥50 | *Crude* | 1.54(1.29~1.84)* | <0.01 | 0.69 | 1.53(1.28~1.82)* | <0.01 | 0.10 | 1.20(1.00~1.44)* | 0.46 | 0.05 |
|  | *Adjusted* | 2.24(1.76~2.86)* | <0.01 | 0.50 | 2.29(1.80~2.92)* | <0.01 | 0.60 | 1.51(1.18~1.94)* | 0.29 | 0.00 |
| Job category (ref= Security guard) | | |  |  |  |  |  |  |  |  |
| Longshoreman | *Crude* | 0.73(0.50~1.05) | 0.09 | 0.07 | 0.69(0.48~1.00)* | 0.05 | 0.14 | 0.61(0.42~0.89)* | 0.45 | 0.01 |
|  | *Adjusted* | 0.92(0.63~1.34) | 0.65 | 0.10 | 0.88(0.60~1.28) | 0.51 | 0.10 | 0.73(0.50~1.08) | 0.47 | 0.12 |
| Skilled worker | *Crude* | 1.93(1.32~2.82)* | <0.01 | 0.52 | 1.78(1.22~2.61)* | <0.01 | 0.29 | 0.90(0.61~1.34) | 0.76 | 0.61 |
|  | *Adjusted* | 1.64(1.10~2.43)* | 0.01 | 0.36 | 1.42(0.96~2.10) | 0.08 | 0.32 | 0.93(0.62~1.39) | 0.62 | 0.71 |
| Driver | *Crude* | 0.84(0.57~1.23) | 0.37 | 0.31 | 0.84(0.57~1.22) | 0.36 | 0.66 | 0.70(0.48~1.05) | 0.77 | 0.08 |
|  | *Adjusted* | 0.87(0.59~1.28) | 0.47 | 0.25 | 0.85(0.58~1.25) | 0.41 | 0.62 | 0.75(0.50~1.11) | 0.68 | 0.15 |
| Inspector | *Crude* | 1.34(0.89~2.03) | 0.17 | 0.49 | 1.14(0.76~1.73) | 0.53 | 0.55 | 1.01(0.66~1.53) | 0.91 | 0.98 |
|  | *Adjusted* | 1.23(0.81~1.88) | 0.34 | 0.29 | 0.98(0.64~1.49) | 0.91 | 0.32 | 1.12(0.73~1.73) | 0.92 | 0.59 |
| Other | *Crude* | 1.55(1.09~2.22)* | 0.02 | 0.41 | 1.53(1.08~2.19)* | 0.02 | 0.18 | 0.94(0.66~1.36) | 0.80 | 0.76 |
|  | *Adjusted* | 1.23(0.85~1.78) | 0.27 | 0.21 | 1.13(0.78~1.63) | 0.51 | 0.12 | 0.98(0.68~1.43) | 0.64 | 0.93 |

Footnotes: *Time_total_*_:_ the answer time of the total population; * *P<0.05* was considered statistically significant; *OR*: odds rate; *95%CI*: 95% confidence interval; *Crude:* no adjustment; *Adjusted*: adjusted for age, sex, education and marriage; ref: reference; The Occupational Health attitude and Occupational Health Behaviors indicators were not included, because their scores did not meet the requirements of quartile; All the ordinal logistic regression models passed the parallel test (*Probability >0.05*).

Table S10 Basic information of participants

(Sensitivity analysis in the population with *Time_total_* range of 1%~99%)

| Characteristics |  | n (%) or Mean ± SD | |
| --- | --- | --- | --- |
| Sample size |  | | 3861 |
| Age |  | | 40.0 (10.1) |
| Sex | Female | | 185 (4.8%) |
|  | Male | | 3676 (95.2%) |
| Education | College degree or above | | 1186 (30.7%) |
|  | Middle or high schools | | 2485 (64.4%) |
|  | Primary school or below | | 190 (4.9%) |
| Marriage | No | | 138 (3.6%) |
|  | Yes | | 2852 (73.9%) |
|  | Other | | 871 (22.6%) |
| Length of service |  | | 15.2 (9.73) |
| Job category | Longshoreman | | 1015 (26.3%) |
|  | Skilled worker | | 517 (13.4%) |
|  | Driver | | 484 (12.5%) |
|  | Inspector | | 255 (6.6%) |
|  | Security guard | | 119 (3.1%) |
|  | Other | | 1471 (38.1%) |
| Occupational Health Literacy | Score | | 52.9 (7.53) |
|  | Yes | | 2780 (72.0%) |
|  | No | | 1081 (28.0%) |
| Occupational Health Knowledges | Score | | 23.5 (4.98) |
|  | Yes | | 2430 (62.9%) |
|  | No | | 1431 (37.1%) |
| Occupational Health Attitude | Score | | 7.29 (1.48) |
|  | Yes | | 3272 (84.7%) |
|  | No | | 589 (15.3%) |
| Occupational Health Behaviors | Score | | 6.78 (0.791) |
|  | Yes | | 3689 (95.5%) |
|  | No | | 172 (4.5%) |
| Occupational Health Skills | Score | | 15.3 (2.09) |
|  | Yes | | 2150 (55.7%) |
|  | No | | 1711 (44.3%) |

Footnotes: *Time_total_*_:_ answer time of the total population; Score threshold of participants who have sufficient occupational health literacy: score of Occupational Health Literacy ≥ 52; score of Occupational Health Knowledges ≥ 24; score of Occupational Health Attitude ≥ 7; score of Occupational Health Behaviors ≥ 6; score of Occupational Health Skills ≥ 16.

Table S11 Relationship between Occupational Health Literacy and length of service or job category based on binary logistic regression

(Sensitivity analysis in the population with *Time_total_* range of 1%~99%)

|  |  | Occupational Health Literacy | | Occupational Health Knowledges | | Occupational Health Attitude | | Occupational Health Behaviors | | Occupational Health Skills | |
| --- | --- | --- | --- | --- | --- | --- | --- | --- | --- | --- | --- |
|  |  | *OR（95%CI）* | *P* | *OR（95%CI）* | *P* | *OR（95%CI）* | *P* | *OR（95%CI）* | *P* | *OR（95%CI）* | *P* |
| Length of service (years, ref= Length of service with ≤32 years) | | | |  |  |  |  |  |  |  |  |
| 33-40 | *Crude* | 1.10(1.05~1.14)* | <0.01 | 1.09(1.04~1.13)* | <0.01 | 1.04(1.01~1.07)* | 0.02 | 1.01(0.99~1.03) | 0.37 | 1.03(0.99~1.08) | 0.16 |
|  | *Adjusted* | 1.12(1.07~1.17)* | <0.01 | 1.11(1.06~1.16)* | <0.01 | 1.04(1.01~1.08)* | 0.01 | 1.01(0.99~1.03) | 0.44 | 1.05(1.01~1.10)* | 0.03 |
| 41-49 | *Crude* | 1.11(1.07~1.16)* | <0.01 | 1.11(1.06~1.16)* | <0.01 | 1.06(1.03~1.10)* | <0.01 | 1.01(0.99~1.03) | 0.16 | 1.08(1.03~1.12)* | <0.01 |
|  | *Adjusted* | 1.14(1.09~1.19)* | <0.01 | 1.13(1.08~1.19)* | <0.01 | 1.08(1.04~1.11)* | <0.01 | 1.01(0.99~1.03) | 0.31 | 1.10(1.05~1.16)* | <0.01 |
| ≥50 | *Crude* | 1.09(1.05~1.13)* | <0.01 | 1.1(1.05~1.15)* | <0.01 | 1.06(1.02~1.09)* | <0.01 | 1.02(1.00~1.04)* | 0.03 | 1.06(1.01~1.10)* | 0.01 |
|  | *Adjusted* | 1.20(1.14~1.26)* | <0.01 | 1.22(1.15~1.29)* | <0.01 | 1.10(1.05~1.14)* | <0.01 | 1.02(0.99~1.04) | 0.13 | 1.13(1.06~1.19)* | <0.01 |
| Job category (ref= Security guard) | | |  |  |  |  |  |  |  |  |  |
| Longshoreman | *Crude* | 0.86(0.79~0.94)* | <0.01 | 0.92(0.84~1.01) | 0.08 | 0.95(0.88~1.01) | 0.11 | 0.98(0.94~1.02) | 0.27 | 0.85(0.77~0.93)* | <0.01 |
|  | *Adjusted* | 0.90(0.83~0.98)* | 0.02 | 0.97(0.89~1.06) | 0.54 | 0.97(0.90~1.04) | 0.40 | 0.97(0.93~1.01) | 0.20 | 0.88(0.80~0.97)* | 0.01 |
| Skilled worker | *Crude* | 1.11(1.01~1.21)* | 0.03 | 1.20(1.09~1.32)* | <0.01 | 1.06(0.99~1.14) | 0.09 | 1.02(0.98~1.07) | 0.25 | 0.97(0.88~1.07) | 0.54 |
|  | *Adjusted* | 1.05(0.96~1.15) | 0.26 | 1.13(1.03~1.24)* | 0.01 | 1.05(0.98~1.13) | 0.20 | 1.02(0.98~1.06) | 0.40 | 0.96(0.86~1.06) | 0.39 |
| Driver | *Crude* | 0.91(0.83~1.00)* | 0.04 | 0.96(0.87~1.06) | 0.41 | 0.94(0.88~1.01) | 0.11 | 0.96(0.92~1.00) | 0.07 | 0.89(0.8~0.98)* | 0.02 |
|  | *Adjusted* | 0.91(0.84~1.00)* | 0.04 | 0.96(0.88~1.06) | 0.44 | 0.95(0.88~1.02) | 0.13 | 0.96(0.92~1.00) | 0.06 | 0.89(0.8~0.98)* | 0.02 |
| Inspector | *Crude* | 1.02(0.92~1.12) | 0.76 | 1.07(0.96~1.19) | 0.21 | 1.00(0.93~1.08) | 0.94 | 0.97(0.93~1.02) | 0.21 | 1.00(0.90~1.12) | 0.94 |
|  | *Adjusted* | 0.98(0.89~1.08) | 0.75 | 1.03(0.93~1.14) | 0.55 | 0.98(0.91~1.06) | 0.65 | 0.97(0.92~1.01) | 0.16 | 1.02(0.92~1.14) | 0.70 |
| Other | *Crude* | 1.04(0.95~1.13) | 0.39 | 1.10(1.01~1.21)* | 0.03 | 1.02(0.96~1.09) | 0.49 | 1.00(0.96~1.04) | 0.85 | 0.97(0.88~1.06) | 0.50 |
|  | *Adjusted* | 0.98(0.90~1.06) | 0.63 | 1.03(0.94~1.12) | 0.54 | 1.00(0.94~1.07) | 0.94 | 0.99(0.95~1.03) | 0.64 | 0.96(0.87~1.06) | 0.42 |

Footnotes: *Time_total_*_:_ answer time of the total population; **P<0.05* was considered statistically significant; *OR*: odds rate; *95%CI*: 95% confidence interval; *Crude:* no adjustment; *Adjusted*: adjusted for age, sex, education and marriage; ref: reference.

Table S12 Relationship between Occupational Health Literacy and the length of service or job category based on ordinal logistic regression

(Sensitivity analysis in the population with *Time_total_* range of 1%~99%)

|  |  | Occupational Health Literacy | | | Occupational Health Knowledges | | | Occupational Health Skills | | |
| --- | --- | --- | --- | --- | --- | --- | --- | --- | --- | --- |
|  |  | *OR（95%CI）* | *P* | *Probability* | *OR（95%CI）* | *P* | *Probability* | *OR（95%CI）* | *P* | *Probability* |
| Length of service (years, ref= Length of service with ≤32 years) | | | |  |  |  |  |  |  |  |
| 33-40 | *Crude* | 1.40(1.19~1.64)* | <0.01 | 0.14 | 1.43(1.22~1.67)* | <0.01 | 0.52 | 1.08(0.91~1.27) | 0.37 | 0.99 |
|  | *Adjusted* | 1.54(1.30~1.82)* | <0.01 | 0.14 | 1.57(1.33~1.87)* | <0.01 | 0.67 | 1.15(0.96~1.37) | 0.12 | 0.98 |
| 41-49 | *Crude* | 1.55(1.32~1.83)* | <0.01 | 0.36 | 1.56(1.33~1.84)* | <0.01 | 0.92 | 1.30(1.10~1.53)* | <0.01 | 0.08 |
|  | *Adjusted* | 1.76(1.46~2.13)* | <0.01 | 0.20 | 1.75(1.45~2.12)* | <0.01 | 0.83 | 1.43(1.18~1.73)* | <0.01 | 0.26 |
| ≥50 | *Crude* | 1.41(1.21~1.65)* | <0.01 | 0.38 | 1.38(1.18~1.62)* | <0.01 | 0.11 | 1.19(1.01~1.40)* | 0.03 | 0.94 |
|  | *Adjusted* | 2.19(1.76~2.72)* | <0.01 | 0.24 | 2.21(1.78~2.74)* | <0.01 | 0.55 | 1.49(1.20~1.86)* | <0.01 | 0.74 |
| Job category (ref= Security guard) | | |  |  |  |  |  |  |  |  |
| Longshoreman | *Crude* | 0.69(0.49~0.97)* | 0.03 | 0.05 | 0.69(0.49~0.97)* | 0.03 | 0.15 | 0.62(0.43~0.88)* | 0.01 | 0.62 |
|  | *Adjusted* | 0.85(0.60~1.21) | 0.38 | 0.04 | 0.86(0.61~1.22) | 0.40 | 0.13 | 0.71(0.49~1.03) | 0.07 | 0.60 |
| Skilled worker | *Crude* | 1.98(1.39~2.83)* | <0.01 | 0.44 | 1.92(1.35~2.74)* | <0.01 | 0.51 | 1.15(0.79~1.67) | 0.47 | 0.13 |
|  | *Adjusted* | 1.64(1.14~2.37)* | 0.01 | 0.22 | 1.50(1.04~2.15)* | 0.03 | 0.60 | 1.09(0.75~1.60) | 0.65 | 0.41 |
| Driver | *Crude* | 0.79(0.55~1.12) | 0.19 | 0.23 | 0.82(0.58~1.17) | 0.28 | 0.67 | 0.80(0.55~1.17) | 0.25 | 0.59 |
|  | *Adjusted* | 0.81(0.56~1.16) | 0.24 | 0.15 | 0.83(0.58~1.19) | 0.31 | 0.60 | 0.81(0.56~1.19) | 0.29 | 0.74 |
| Inspector | *Crude* | 1.34(0.91~1.97) | 0.14 | 0.29 | 1.18(0.80~1.73) | 0.41 | 0.44 | 1.12(0.74~1.68) | 0.60 | 0.68 |
|  | *Adjusted* | 1.20(0.81~1.79) | 0.36 | 0.15 | 0.97(0.65~1.44) | 0.88 | 0.29 | 1.15(0.76~1.74) | 0.51 | 0.70 |
| Other | *Crude* | 1.51(1.09~2.11)* | 0.01 | 0.24 | 1.56(1.12~2.17)* | 0.01 | 0.22 | 1.09(0.77~1.56) | 0.61 | 0.80 |
|  | *Adjusted* | 1.19(0.84~1.67) | 0.32 | 0.09 | 1.14(0.81~1.60) | 0.46 | 0.16 | 1.05(0.73~1.51) | 0.78 | 0.93 |

Footnotes: *Time_total_*_:_ the answer time of the total population; * *P<0.05* was considered statistically significant; *OR*: odds rate; *95%CI*: 95% confidence interval; *Crude:* no adjustment; *Adjusted*: adjusted for age, sex, education and marriage; ref: reference; The Occupational Health attitude and Occupational Health Behaviors indicators were not included, because their scores did not meet the requirements of quartile; All the ordinal logistic regression models passed the parallel test (*Probability >0.05*).

Figure S3. Occupational protection pattern implemented among port employees


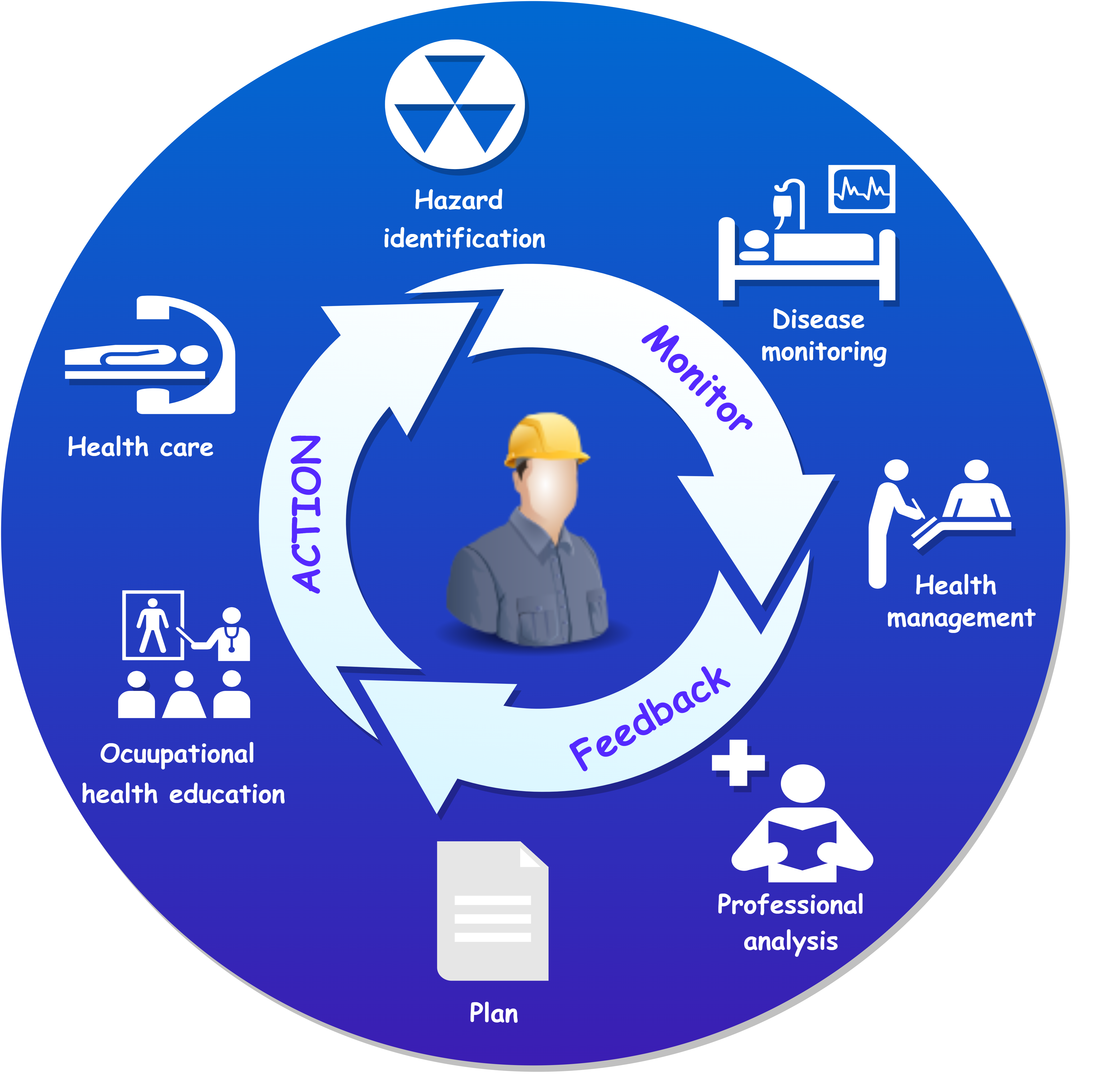


**Questionnaire 1: Occupational Health Literacy Questionnaire**

(1) Occupational health knowledge (please tick "√" based on your actual situation)

- 1. . What do you think "health" means? [Single choice]

🞎 Health means physical health without disease.

🞎 Health means mental health and physical health.

🞎 Health is not only physical health but also the well-being of psychological health and social adaptation.

🞎 Not clear.

1.2. Which lifestyle do you think will increase occupational hazards? [Multiple choice]

🞎 Lacking exercise

🞎 Being optimistic

🞎 Smoking and drinking

🞎 Not clear

1.3. What do you think is greatly affected by long-term overtime work and shift work? [Multiple choice]

🞎 Affected physical health

🞎 Affected mental health

🞎 Reduced work efficiency

🞎 Unclear

1.4. Which one of the following choices is best to prevent heat stroke after sweating in hot weather? [Multiple choice]

🞎 Milk

🞎 Soft drinks containing salt and water-soluble vitamins

🞎 Ice water

🞎 Unclear

1.5. Which one is correct about the use of protective equipment? [Multiple choice]

🞎 Using inappropriate personal protective equipment is not only ineffective but also may be harmful.

🞎 Personal protective equipment should be changed regularly.

🞎 If the toxic concentration in working place is lower than the national standard limit, it is not necessary to use a respirator.

🞎 Unclear

1.6. In your opinion, what are the benefits of occupational health examination? (Multiple choice)

🞎 Estimate whether or not you are suitable for the job.

🞎 Find work-related health damage as soon as possible.

🞎 Get economic compensation

🞎 Unclear

1.7. Which of the following diseases are legal occupational diseases in China? (Reserved) [multiple choice]

🞎 Lumbar Disc Herniation of Drivers

🞎 Pneumoconiosis in coal miners

🞎 Toxic liver disease in workers who use toxic chemicals

🞎 Unclear

1.8. When you encounter someone with sudden respiratory or cardiac arrest, which measure is suitable to take? [Multiple choice]

🞎 CPR (cardio-pulmonary resuscitation)

🞎 Call 120

🞎 Give hypertension medication

🞎 Unclear

1.9. Which one of the following do you think is an occupational musculoskeletal disorder? [Multiple choice]

🞎 Bus driver's neck and shoulder pain caused by sitting for a long time

🞎 Video operator's "mouse hand"

🞎 Courier's lower back pain caused by handling goods

🞎 Unclear

**（2）Occupational health attitude (please tick "√" based on your actual situation)**

2.1. Occupational and work-related diseases can be prevented, and prevention is more important than cure. [Single choice]

🞎 Disagree

🞎 Agree

🞎 Unclear

2.2. It is not necessary to attend the occupational health training organized by the employer, because it is too time-consuming. [Single choice]

🞎 Disagree

🞎 Agree

🞎 Unclear

2.3. It will not affect my health even if hiding something in the Orientation Health Check to get a job. [Single choice]

🞎 Disagree

🞎 Agree

🞎 Unclear

2.4. Personal protective equipment is the last line of defense to prevent exposure to occupational hazards. Although sometimes wearing personal protective equipment may be a little uncomfortable, we also should keep wearing them. [Single choice]

🞎 Disagree

🞎 Agree

🞎 Unclear

2.5. Medical workers, sanitation workers, and other special occupational groups may be prone to infect or transmit diseases, hence, they should protect more against infectious diseases. [Single choice]

🞎 disagree

🞎 agree

🞎 Unclear

2.6. Maintaining good mental health at work can help reduce the risk of occupational and work-related diseases. [Multiple choice]

🞎 Disagree

🞎 Agree

🞎 Unclear

2.7. Workers engaged in toxic or harmful jobs are entitled to occupational protection. [Multiple choice]

🞎 Disagree

🞎 Agree

🞎 Unclear

2.8. Safeguarding the occupational health of workers is a common responsibility of the government, employers, and workers, but workers should be responsible for their health. [Single choice]

🞎 Disagree

🞎 Agree

🞎 Unclear

**（3）Occupational health behavior (Please tick "√" based on your actual situation)**

3.1. Take the initiative to know the occupational hazards. [Single choice]

🞎 Never

🞎 Occasionally

🞎 Often

🞎 Always

3.2. Participate in occupational health training organized by the company before and during employment. [Single choice]

🞎 Never

🞎 Occasionally

🞎 Often

🞎 Always

3.3. Pay attention to your physical health and get regular health examinations. [Single choice]

🞎 Never

🞎 Occasionally

🞎 Often

🞎 Always

3.4. Strictly comply with the job operation specifications, keep ventilation, disinfect the workplace or check and open the protective equipment regularly following the requirements. [Single choice]

🞎 Never

🞎 Occasionally

🞎 Often

🞎 Always

3.5. Wash hands before drinking or eating during the working break. [Single choice]

🞎 Never

🞎 Occasionally

🞎 Often

🞎 Always

3.6. I take breaks (such as breaks for drinking water or doing work-break exercises) when working for a long time. [Single choice]

🞎 Never

🞎 Occasionally

🞎 Often

🞎 Always

3.7. Open a window for ventilation to dilute the bacteria and viruses. [Single choice]

🞎 Never

🞎 Occasionally

🞎 Often

🞎 Always

**(iv) Occupational Health Skills (please tick "√" in the corresponding box)**

4.1.1. What do you think the following warning sign represents?
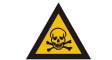
 [Single choice]

🞎 Flammable

🞎 High pressure

🞎 Radioactivity

🞎 Biosafety

🞎 Explosive

🞎 Highly toxic

🞎 Lightning

🞎 Unknown

4.1.2. What do you think the following warning sign means?
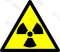
[Multiple choice]

🞎 Flammable

🞎 High pressure

🞎 Radioactivity

🞎 Biosafety

🞎 Explosive

🞎 highly toxic

🞎 Lightning

🞎 unknown

4.1.3. What do you think the following warning sign means?
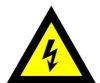
[Single choice]

🞎 Flammable

🞎 High voltage

🞎 Radioactive

🞎 Biosafety

🞎 Explosive

🞎 Highly toxic

🞎 Lightning

🞎unclear

4.1.4. What do you think the following warning sign means?
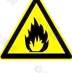
[Single choice]

🞎 Flammable

🞎 High pressure

🞎 Radioactivity

🞎 Biosafety

🞎 Explosive

🞎Highly toxic

🞎 Lightning

🞎 Unknown

4.2. What measures do you take if the toxic liquid is spilled or accidentally splashed on your skin? [Multiple choice]

🞎 Do nothing

🞎 Wipe the liquid with a rag

🞎 Wash the affected area with lots of water

🞎 Put the rag into a closed garbage bin

🞎 Unclear

4.4. Which emergency numbers can you call in case of an accident? [Multiple choice]

🞎 119

🞎 120

🞎 110

🞎 114

🞎 911

4.5. What first aid measures would you take in case of toxic chemical poisoning? [multiple choice]

🞎 quickly escape from the hazard

🞎 Remove contaminated clothing

🞎 Turn off the emergency switch, sound the alarm

🞎 Rescue workmates as soon as possible

🞎 Unclear
